# High-Resolution Optical Coherence Tomography Retinal Imaging: A Case Series Illustrating Potential and Limitations

**DOI:** 10.1155/2011/764183

**Published:** 2011-09-29

**Authors:** Olena Puzyeyeva, Wai Ching Lam, John G. Flanagan, Michael H. Brent, Robert G. Devenyi, Mark S. Mandelcorn, Tien Wong, Christopher Hudson

**Affiliations:** ^1^Retina Research Group, Department of Ophthalmology and Vision Sciences, University of Toronto, Toronto, ON, M5T 2S8, Canada; ^2^School of Optometry, University of Waterloo, Waterloo, ON, N2L 3G1, Canada

## Abstract

*Purpose*. To present a series of retinal disease cases that were imaged by spectral domain optical coherence tomography (SD-OCT) in order to illustrate the potential and limitations of this new imaging modality. *Methods*. The series comprised four selected cases (one case each) of age-related macular degeneration (ARMD), diabetic retinopathy (DR), central retinal artery occlusion (CRAO), and branch retinal vein occlusion (BRVO). Patients were imaged using the Heidelberg Spectralis (Heidelberg Engineering, Germany) in SD-OCT mode. Patients also underwent digital fundus photography and clinical assessment. *Results*. SD-OCT imaging of a case of age-related macular degeneration revealed a subfoveal choroidal neovascular membrane with detachment of the retinal pigment epithelium (RPE) and neurosensory retina. Using SD-OCT, the cases of DR and BRVO both exhibited macular edema with cystoid spaces visible in the outer retina. *Conclusions*. The ability of SD-OCT to clearly and objectively elucidate subtle morphological changes within the retinal layers provides information that can be used to formulate diagnoses with greater confidence.

## 1. Introduction

Over the past decade, the development of high-speed, wide-bandwidth tuneable light sources in conjunction with high-speed photodetectors has resulted in major gains in the horizontal and depth resolution of optical coherence tomography (OCT) based instrumentation, thereby dramatically improving visualization capabilities during retinal and optic nerve examination [1, 2]. As a result, OCT has found its place as a widely accepted imaging technique, especially in ophthalmology. 

The principle of OCT is based on interferometry [3, 4]. In a typical early generation OCT system, visible light (i.e., to visualise the beam) and broadband, short-coherence length, near-IR light are coupled into one branch of a Michelson interferometer. The light is then split into two paths, one leading to a reference mirror and the second is focused onto the retina. Light is reflected and backscattered from refractive index interfaces within the retina according to the optical properties of each interface. The reflected light from the retina (i.e., the sample arm) and from the reference mirror is recoupled into the interferometer, to ultimately be detected after interference in the spectrometer. Using “time domain” OCT, reflection sites at various depths in the tissue can be sampled by changing the path length of the reference arm. However, this mechanism limits the acquisition speed (approx. 400 A-scans/s), is prone to motion artifacts because of the slow scan speed, and makes real-time imaging impossible. The reference-arm mirror is also scanned at a constant velocity, allowing depth scans to be made pixel by pixel across the retina. 

**Figure 1:**
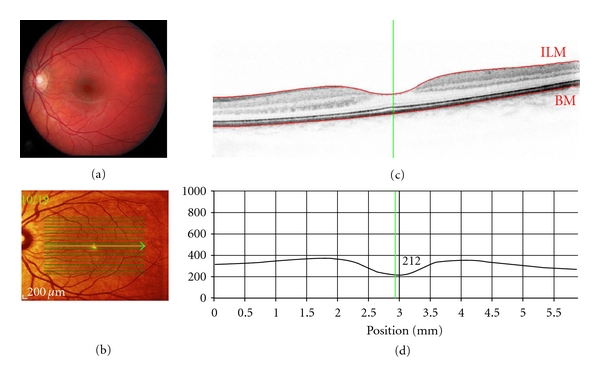
Spectral-domain (SD-OCT) optical coherence tomography of a healthy retina (OS): Scan parameters: infrared scan angle 30°; OCT scan angle 20°; pattern size 20°×15°, 19 sections (244 *μ*m between B-scans). (a) Conventional fundus camera image. (b) SD-OCT en-face image showing overlaid OCT scan lines (green) and scan area. The green arrow shows the position of the scan line used to generate the cross-sectional retinal OCT image (i.e., (c)). (c) Cross-sectional image of the retina depicts the vitreous cavity (upper, optically clear area), the internal limiting membrane (segmented by the upper red line marked ILM, the intervening retinal layers, Bruch's membrane which is segmented by the lower red line marked BM, and the underlying choroid (lower). The vertical green line defines the position of the retinal thickness measure along the cross-sectional retinal thickness profile. (d) Cross-sectional retinal thickness profile corrected for tilt.

SD-OCT has dramatically improved image resolution. Using SD-OCT, broadband interference is measured with spectrally distinct detectors using Fourier analysis (i.e., light signal frequency is modulated as a function of depth), thereby avoiding path length adjustment of the reference arm. The avoidance of depth scanning results in dramatic gains in imaging speed (i.e., 20,000 to 40,000 A-scans/s) and improved signal-to-noise ratio [5], with an axial resolution of approximately 7 *μ*m, thereby permitting the acquisition of high-resolution, histological detail of the retina captured from the living human eye over a wide field of view. The much improved scan speed of SD-OCT also permits 3D scanning, with minimal impact of eye movements. The SD-OCT scans can also be referenced to simultaneously acquired 2D en-face images, thereby ensuring the accurate spatial location of each OCT A-scan within the 3D image. An example of SD-OCT imaging of a healthy retina is shown in Figure 1. 

In this study, we report on the clinical application of SD-OCT using a series of case reports of patients with clinically defined common and/or classic eye diseases in order to highlight some of the potential, the limitations, and the clinical utility of this technology.

## 2. Methods

Patients were imaged using the Heidelberg Spectralis HRA + OCT (Heidelberg Engineering, Heidelberg, Germany) in SD-OCT mode, using a scan field of 30 degrees horizontally and 15 degrees vertically and 19 to 25 OCT horizontal sections (one section at least every 240 *μ*m). Patients also underwent pupil dilation, digital fundus photography, and clinical assessment. Digital fundus photography was undertaken using a Canon digital fundus camera (Canon CR-DGi, Canon Inc., Japan) with a resolution of 12.8 mega pixels. Clinical assessment comprised visual acuity, stereo fundus biomicroscopy, and binocular indirect ophthalmoscopy, as appropriate.

### 2.1. The Heidelberg Spectralis HRA and OCT

The Heidelberg Spectralis HRA and OCT (Heidelberg Engineering, Heidelberg, Germany; Software version-1.6.1.0) can be used in any one of six imaging modes, that is, SD-OCT, fluorescein angiography, indocyanine green angiography, autofluorescence, and red-free and infrared imaging. This paper details use of the instrument in SD-OCT mode only. The Heidelberg Spectralis utilizes a broadband light source centered at 870 nm (i.e., no visible light “beacon”) to simultaneously measure multiple wavelengths, a prerequisite of SD-OCT imaging (Heidelberg Retina Angiograph 2 Operating Instructions). Simultaneous confocal scanning laser ophthalmoscopy is used to generate high-resolution images of the retinal surface, thereby providing precise location information of each A-scan within a cross-sectional SD-OCT image. SD-OCT scanning generates 40,000 A-scans/second with an axial resolution of 3.5 microns/pixel digital (7 microns optical) and a transverse resolution of 14 microns [6]. Alignment software continuously tracks any eye movement during image acquisition and then adjusts the position of the A-scan on the retinal surface to ensure accurate registration of cross-sectional OCT images. Using eye tracking and registration technology, multiple images are obtained from a precise location to then be averaged and filtered to remove random noise from the final image. The same eye tracking/registration technology is used to ensure that the instrument automatically rescans images that are influenced by blink artifacts. Similarly, follow-up images are derived from the same area of retina, thereby eliminating subjective placement of the scan by the operator.

## 3. Case Reports

This series comprised four selected cases (one case each) of age-related macular degeneration (ARMD), diabetic retinopathy (DR), central retinal artery occlusion (CRAO), and branch retinal vein occlusion (BRVO). 

### 3.1. Case  1: Exudative Age-Related Macular Degeneration (ARMD) (Figure 2)

A 81-year-old female patient had a 20-year history of hypertension and a one-year history of type 2 diabetes. At first presentation, her best corrected visual acuity (VA) in the right (OD) and left (OS) eyes was 20/50 and 20/70, respectively. Intraocular pressures (IOPs) were 18 mmHg OD and 20 mmHg OS. Retinal examination revealed a large choroidal neovascular membrane (CNVM) and a probable serous pigment epithelium detachment (PED) OD and soft macular drusen OS (not shown). 

### 3.2. Case  2: Treated Proliferative Diabetic Retinopathy with Clinically Significant Diabetic Macular Edema (Figure 3)

A 51-year-old male patient presented with a 15 year history of type 2 diabetes, having taken oral medications for the first 11 years and having used insulin for the past 4 years. Past ocular history included laser photocoagulation in both eyes. At the initial visit, the best corrected visual acuity was 20/30 (OD) and 20/70 (OS) with IOPs of 20 mmHg OD and OS. 

### 3.3. Case  3: Central Retinal Artery Occlusion (Figure 4)

A 69-year-old male patient had a medical history of stroke and type 2 diabetes for fifteen years. He presented in the clinic with sudden and absolute vision loss OD. Visual acuity was Counting Fingers at 0.3 meters OD and 20/40 OS. 

### 3.4. Case  4: Branch Retinal Vein Occlusion (Figure 5)

A 78-year-old male patient had a 10 year history of hypertension. His past ocular history included cataract surgery to both eyes. The patient complained of blurry vision OS for the past 4 months. At the initial visit, the visual acuity was Counting Fingers at 0.07 meters OD and 20/200 OS with an intraocular pressure of 24 mmHg and 18 mmHg. 

## 4. Discussion

SD-OCT imaging technology was used to acquire images of patients with various retinal diseases in order to evaluate the clinical utility, potential, and limitations of the technique. Both SD-OCT and conventional clinical techniques showed choroidal neovascular membrane and pigment epithelial detachment (case of ARMD); neovascularisation at the disc and elsewhere, fibrosis, epiretinal membrane, and laser scars (case of DR); retinal edema and haemorrhages (case of BRVO). In some circumstances, SD-OCT provided visualization of morphological changes associated with retinal diseases that were either not immediately visible or not at all visible, using conventional clinical techniques. For example, SD-OCT revealed neurosensory retinal detachment and Bruch's/retinal pigment epithelium wrinkling (case of ARMD); cystoid spaces localised in the outer retina (case of DR); thickening and increased reflectance of inner retina (case of CRAO); localisation of depth of macular edema and of haemorrhages (case of BRVO). Thus, SD-OCT revealed structural retinal changes that are not visible by 2-dimensional limited fundus photography. Conversely, the presence of colour information within the digital fundus photography images may be advantageous, while SD-OCT uses a narrow spectrum of wavelengths and therefore has limited colour information. For example, in the case of the CRAO, conventional digital fundus photography showed the presence of infarction more prominently than the cSLO and SD-OCT imaging.

Previous studies have shown that SD-OCT reveals retinal pathology that was not visible using TD-OCT, such as intra-retinal cysts and subretinal fluid [7]. SD-OCT also adds information to complete the clinical picture providing more detailed resolution of retinal changes, such as full-thickness folds of the RPE and intraretinal edema in case of RPE detachment, BRVO, toxoplasma chorioretinitis, and polypoidal choroidal vasculopathy [8]. A feature of the Heidelberg Spectralis instrument is the ability to undertake multiple mode imaging in addition to SD-OCT, including fluorescein angiography, indocyanine green angiography and autofluorescence, and red-free and infrared imaging. These additional imaging modes also provide further information about retinal pathology that can aid diagnosis and management. A description of these imaging modes is provided elsewhere [9, 10].

The ability of SD-OCT to clearly and objectively elucidate subtle morphological changes within the retinal layers provides information that could potentially be useful in the treatment of retinal diseases. This feature provides SD-OCT with a clear superiority over other clinical techniques that do not possess the same resolution. First, high-resolution cross-sectional images allow better visualization of the vitreoretinal interface, the vitreous, retinal structures, and the choroid. Furthermore, 3D images depict volumetric topographic retinal morphology that can be registered relative to images acquired at a different time point and, therefore, change in retinal morphology can be calculated. Second, while taking a follow-up image of a particular patient, the software is capable of automatically and accurately registering images so that the identical retinal area is used to calculate change. This added functionality eliminates the possibility of human error and makes it easier to analyze the data with greater validity. However, automated segmentation of the internal limiting membrane and Bruch's membrane sometimes requires manual adjustment prior to analysis in patients with retinal diseases.

Nevertheless, SD-OCT has its limitations. This paper clearly demonstrates that hyperreflective lesions such as exudates and haemorrhages, as well as major retinal vessels, resulted in shadowing of the underlying retinal structures, and thereby details of the underlying morphology are lost. In the case exhibiting choroidal neovascular membrane (i.e., Case  1), and diabetic retinopathy/macular edema where the retinal thickness was over 400 *μ*m, it was hard to discern the underlying pathology and choroid.

## 5. Conclusion

 SD-OCT imaging technology offers a previously unattainable resolution of retinal morphology. The ability of SD-OCT to clearly and objectively elucidate subtle morphological changes within the retinal layers provides information that can be used to potentially formulate diagnoses earlier and with greater confidence. The current generation of SD-OCT instruments will not replace clinical retinal evaluation but do offer further information that can be valuable from a clinical perspective.

##  Statement Summary

This study illustrates the potential and limitations of SD-OCT imaging technology. It demonstrated the ability of SD-OCT to clearly and objectively elucidate subtle morphological changes within the retinal layers that are not visible using conventional clinical techniques. Such information may be useful for the earlier diagnosis and treatment of retinal diseases.

## Figures and Tables

**Figure 2:**
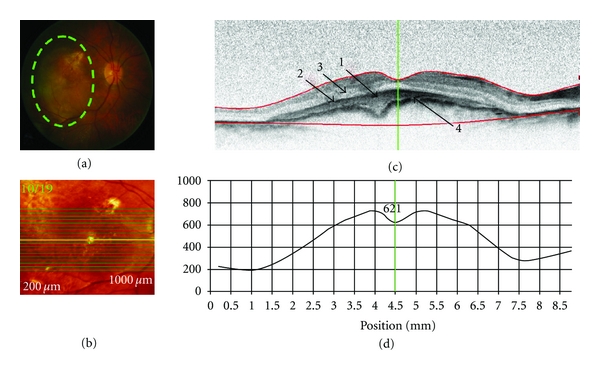
SD-OCT retinal scan of a patient with exudative age-related macular degeneration (OD). Scan parameters: infrared scan angle 30°; OCT scan angle 30°; pattern size 30°×15°, 19 sections (244 *μ*m between B-scans). (a) The conventional fundus camera image showed a large choroidal neovascular membrane (CNVM) situated beneath the macula with a probable serous pigment epithelium detachment (PED). (b) SD-OCT en-face image centered on the fovea. The green highlighted line shows the position of the scan line used to generate the cross-sectional retinal OCT image (i.e., (c)). (c) The cross-sectional retinal image revealed a subfoveal CNVM (arrow “1”) with a pigment epithelial detachment (PED) (arrow “2”) and possible neurosensory retinal detachment (arrow “3”) with apparent thickening and wrinkling of the Bruch's membrane/RPE complex. (d) Cross-sectional retinal thickness profile revealed increased thickness of the retina due to the CNVM. The lower red segment line (shown in (c)) fails to fit the true position of Bruch's membrane (arrow “4”) in the area of the CNVM.

**Figure 3:**
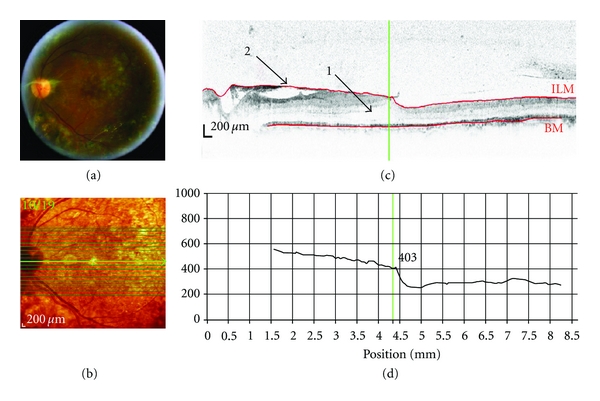
SD-OCT of a patient with proliferative diabetic retinopathy and clinically significant diabetic macular edema (DME) (OS). Scan parameters: infrared scan angle 30°; OCT scan angle 30°; pattern size 30°×15°, 19 sections (240 *μ*m between B-scans). (a) The conventional fundus camera image showed clinically significant DME, fibrous tissue/epiretinal membrane temporal to the optic nerve head (ONH) extending towards the fovea and inferior to the fovea, neovascularisation at the ONH and also at the inferior macula and laser scars in the macula area and outside of the major retinal arcades. (b) SD-OCT en-face image centered on the fovea. The green arrow shows the position of the scan line used to generate the cross-sectional retinal OCT image (i.e., (c)). (c) The cross-sectional retinal image revealed cystoid spaces temporal to the ONH and extending close to the fovea in the outer retina (arrow “1”). The fibrous tissue/epiretinalmembrane can also be seen, located between the ONH and fovea ( arrow “2”). (d) The cross-sectional retinal thickness profile revealed increased retinal thickness especially nasally to the fovea.

**Figure 4:**
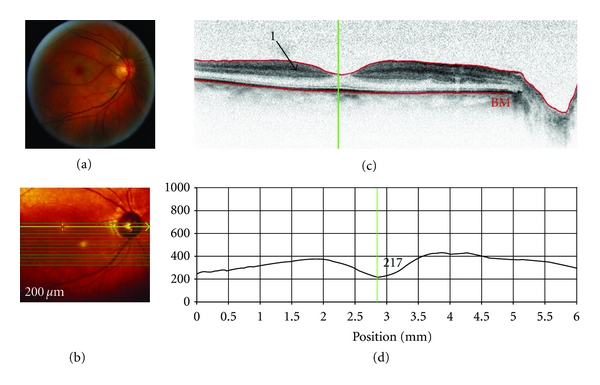
SD-OCT of a patient with central retinal artery occlusion (OD). Scan parameters: infrared scan angle 30°; OCT scan angle 30°; pattern size 30°×10°, 13 sections (243 *μ*m between B-scans). (a) The conventional fundus camera image revealed a classic “cherry red spot” and white infarctions (ischemic areas) along the major vessel arcades and around the macula. (b) SD-OCT en-face image centered approximately 2° below the fovea. The green arrow shows the position of the scan line used to generate the cross-sectional retinal OCT image (i.e., (c)). (c) The cross-sectional retinal image showed a thickening and increased reflectance (arrow “1”) of the inner retinal layers. (d) The cross-sectional retinal thickness profile revealed increased retinal thickness that was especially apparent as an exaggerated foveal pit (indicating swelling of the parafoveal retina).

**Figure 5:**
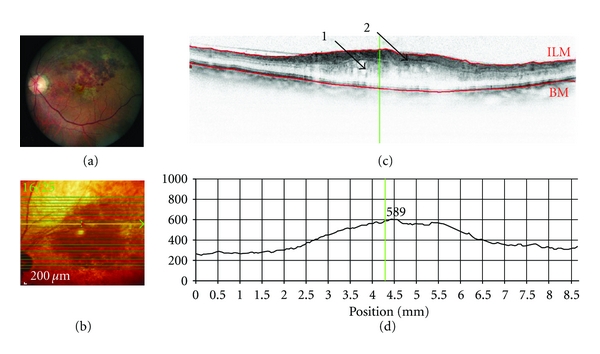
SD-OCT of a patient with branch retinal vein occlusion (OS). Scan parameters: infrared scan angle 30°; OCT scan angle 30°; pattern size 30°×20°, 25 sections (243 *μ*m between B-scans). (a) The conventional fundus camera imaging revealed numerous hemorrhages and edema in the superior temporal retina with macular involvement. The macula was yellow and edematous. (b) SD-OCT en-face image of the fundus (note that the cross-sectional image is located approximately 5° superior to the superior ONH margin). The green arrow shows the position of the scan line used to generate the cross-sectional retinal OCT image (i.e., (c)). (c) The cross-sectional retinal image showed retinal edema with fluid cysts in the outer retina (arrow “1”) and an *apparently *thickened inner retina with superficial hemorrhages (arrow “2”) which “shadow” the underlying retinal details. (d) The lower red segment line (shown in (c)) fails to fit the true position of Bruch's membrane; the line was positioned based on visible parts of Bruch's membrane. The cross-sectional retinal thickness profile revealed a markedly thickened retina in the center of the BRVO (retinal thickness is close to 600 *μ*m).
